# Supplementary material for: Detection of Selected Canine Viruses in Nigerian Free-Ranging Dogs Traded for Meat Consumption
Source: Animals (Basel). 2023 Mar 22;13(6):1119. doi: 10.3390/ani13061119 (PMC10044693; doi:10.3390/ani13061119)
Supplement: Supplementary file 1 [file animals-13-01119-s001.zip › animals-2199098-supplementary.docx]

**Supplementary Materials**

Table S1. Oligonucleotides used in this study.

| Assay | Oligonucleotide | Sequence | Pathogen | References |
| --- | --- | --- | --- | --- |
| Real-time PCR | CAVF | 5′-AGTAATGGAAACCTAGGGG-3′ | CAdV-1/2 | [36] |
|  | CAVR | 5′-TCTGTGTTTCTGTCTTGC-3′ | CAdV-1/2 |  |
|  | CAV1-Pb | FAM-TCAATCGTCTCAACTAAATGCCGTG-BHQ1 | CAdV-1 |  |
|  | CAV2-Pb | TxR-TCAGTCATCTCAGCTCAATGCCGTG-BHQ1 | CAdV-2 |  |
| Real-time PCR | CPV-For | 5′-AAACAGGAATTAACTATACTAATATATTTA-3′ | CCPV-1 | [34] |
|  | CPV-Rev | 5′-AAATTTGACCATTTGGATAAACT-3′ | CCPV-1 |  |
|  | CPV-Pb | FAM –TGGTCCTTTAACTGCATTAAATAATGTACC - TAMRA | CCPV-1 |  |
| Real-time PCR | FPV/CPV-For | 5′-ACAAGATAAAAGACGTGGTGTAACTCAA-3′ | CPV/FPV | [38] |
|  | FPV/CPV-Rev | 5′-CAACCTCAGCTGGTCTCATAATAGT-3′ | CPV/FPV |  |
|  | FPV-Pb | VIC – ATGGGAAATACAGACTATAT - MGB | FPV |  |
|  | CPV-Pb | FAM – ATGGGAAATACAAACTATAT - MGB | CPV |  |
| Real-time PCR | CPVa/b-For | 5′-AGGAAGATATCCAGAAGGAGATTGGA-3′ | CPV-2a/  CPV-2b | [35] |
|  | CPVa/b-Rev | 5′-CCAATTGGATCTGTTGGTAGCAATACA-3′ | CPV-2a/  CPV-2b |  |
|  | CPVa-Pb | VIC – CTTCCTGTAACAAATGATA – MGB | CPV-2a |  |
|  | CPVb1-Pb | FAM – CTTCCTGTAACAGATGATA – MGB | CPV-2b |  |
| Real-time PCR | CPVb/c-For | 5′-GAAGATATCCAGAAGGAGATTGGATTCA-3′ | CPV-2b/  CPV-2c | [35] |
|  | CPVb/c-Rev | 5′-ATGCAGTTAAAGGACCATAAGTATTAAATATATTAGTATAGTTAATTC-3′ | CPV-2b/  CPV-2c |  |
|  | CPVb2-Pb | FAM – CCTGTAACAGATGATAAT - MGB | CPV-2b |  |
|  | CPVc-Pb | VIC – CCTGTAACAGAAGATAAT - MGB | CPV-2c |  |
| PCR | CPV-2679F | 5′-CCAGATCATCCATCAACATCA-3′ | CPV/FPV | [38] |
|  | CPV-3511R | 5′-TGAACATCATCTGGATCTGTACC-3′ | CPV/FPV |  |
|  | F1 | 5′-AGATAGTAATAATACTATGCCATTT-3′ | CPV/FPV | [39] |
|  | R3 | 5′-CCTATATCAAATACAAGTACAATA-3′ | CPV/FPV |  |
| Real-time PCR | DogCV-forward | 5′-CTTGCGAGAGCTGCTCCTTATAT-3′ | CanineCV | [14] |
|  | DogCV-reverse | 5′-CTCCACTTCCGTCTTCCAGTTC-3′ | CanineCV |  |
|  | DogCV-probe | TCCGGAGATGACCACGCCCC | CanineCV |  |
| First PCR | CV-F1 | 5′-GGIAYICCICAYYTICARGG-3′ | Circoviruses | [40] |
|  | CV-R1 | 5′-AWCCAICCRTARAARTCRTC-3′ | Circoviruses |  |
| nested PCR | CV-F2 | 5′-GGIAYICCICAYYTICARGGITT-3′ | Circoviruses |  |
|  | CV-R2 | 5′-TGYTGYTCRTAICCRTCCCACCA-3′ | Circoviruses |  |
| Real-time PCR | CDV-F | 5′-AGCTAGTTTCATCTTAACTATCAAATT-3 | CDV | [37] |
|  | CDV-R | 5′-TTAACTCTCCAGAAAACTCATGC-3′ | CDV |  |
|  | CDV-Pb | FAM-ACCCAAGAGCCGGATACATAGTTTCAATGC-TAMRA | CDV |  |
| RT-PCR | P5 | 5′-CAAAGACGTGTGGTCGGAGAA-3′ | CDV | [41] |
|  | H5F | 5′-GGACAGTTGCCATCTTACGG-3′ | CDV |  |

CCPV-1, Carnivore protoparvovirus-1; CPV, canine parvovirus; FPV, feline panleukopenia virus; CAdV-1, canine adenovirus type 1; CAdV-2, canine adenovirus type 2; CDV, canine distemper virus; CanineCV, canine circovirus; RT, reverse transcription

Table S2. Summary table of nucleotide (nt) and amino acid (aa) substitutions (in bold) in the VP2 region of canine parvoviruses detected in blood samples of dogs in Nigeria (underlined strains in bold) as compared to Nigerian reference strains used for the phylogeny.

|  |  | aa position (aa mutation) (nt position) | | | | | | | | | | | | | | | |
| --- | --- | --- | --- | --- | --- | --- | --- | --- | --- | --- | --- | --- | --- | --- | --- | --- | --- |
| CPV | Strain-  GenBank Accession nr | 5 (Gly →Ala)  (13-15) | 226 (Gly→ Ser)  (676-678) | 229 (Ala→ Pro)  (685-687) | 350 (His→ Gln)  (1048-1050) | 365 (His →Gln)  1093-1095 | 370(Arg→ Gln)  (1108-1110) | 382 (Thr →Arg)  (1144-1146) | 424 (Ala→Val)  (1270-1272) | 440 (Ala→Thr)  (1318-1320) | 499 (Ile →Phe)  (1495-1497) | 520 (Gly→Arg)  (1558-1560) | 558 (Pro→Gln)  (1672-1674) | 560 (Thr→Asn)  (1678-1680) | 565 (Lys→Asn)  (1693-1695) | 582 (Asn→Lys)  (1744-1746) | 584 (Phe→Tyr)  (1751-1753) |
| 2a | NGA/2010/dog/3-10NGR-  HQ602990 | Ala (GCA) | Ser (AGT) | Pro (CCA) | Gln (CAA) | Gln (CAA) | Gln (CAA) | Arg (AGA) | Val (GTA) | **Ala** (**G**CA) | Phe (TTT) | Arg (AGA) | Gln (CAA) | Asn (AAC) | Asn (AAT) | Lys (AAA) | Tyr (TAC) |
| 2a | NGA/2010/dog/1-10NGR-  HQ602991 | Ala (GCA) | Ser (AGT) | Pro (CCA) | Gln (CAA) | Gln (CAA) | Gln (CAA) | Arg (AGA) | Val (GTA) | Thr (ACA) | Phe (TTT) | Arg (AGA) | Gln (CAA) | Asn (AAC) | Asn (AAT) | Lys (AAA) | Tyr (TAC) |
| 2a | NGA/2010/dog/19-10NGR-  HQ602992 | Ala (GCA) | Ser (AGT) | Pro (CCA) | Gln (CAA) | Gln (CAA) | Gln (CAA) | Arg (AGA) | Val (GTA) | Thr (ACA) | Phe (TTT) | Arg (AGA) | Gln (CAA) | Asn (AAC) | Asn (AAT) | Lys (AAA) | Tyr (TAC) |
| 2a | NGA/2010/dog/12-10NGR-  HQ602993 | Ala (GCA) | Ser (AGT) | Pro (CCA) | Gln (CAA) | Gln (CAA) | Gln (CAA) | Arg (AGA) | Val (GTA) | Thr (ACA) | Phe (TTT) | Arg (AGA) | Gln (CAA) | Asn (AAC) | Asn (AAT) | Lys (AAA) | Tyr (TAC) |
| 2a | NGA/2010/dog/23-10NGR-  HQ602994 | Ala (GCA) | Ser (AGT) | Pro (CCA) | Gln (CAA) | Gln (CAA) | Gln (CAA) | Arg (AGA) | Val (GTA) | Thr (ACA) | Phe (TTT) | Arg (AGA) | Gln (CAA) | Asn (AAC) | Asn (AAT) | Lys (AAA) | Tyr (TAC) |
| 2a | NGA/2010/dog/15-10NGR-  HQ602995 | Ala (GCA) | Ser (AGT) | Pro (CCA) | Gln (CAA) | Gln (CAA) | Gln (CAA) | Arg (AGA) | Val (GTA) | Thr (ACA) | Phe (TTT) | Arg (AGA) | Gln (CAA) | Asn (AAC) | Asn (AAT) | Lys (AAA) | Tyr (TAC) |
| 2a | NGA/2018/dog/IZSSI_PA1464/19_idUV1-  MK895483 | Ala (GCA) | Ser (AGT) | Pro (CCA) | Gln (CAA) | Gln (CAA) | Gln (CAA) | Arg (AGA) | Val (GTA) | **Ala** (**G**CA) | Phe (TTT) | Arg (AGA) | Gln (CAA) | Asn (AAC) | Asn (AAT) | Lys (AAA) | Tyr (TAT) |
| 2a | NGA/2018/dog/IZSSI_PA1464/19_idYV8-  MK895484 | Ala (GCA) | Ser (AGT) | Pro (CCA) | Gln (CAA) | Gln (CAA) | Gln (CAA) | Arg (AGA) | Val (GTA) | **Ala** (**G**CA) | Phe (TTT) | Arg (AGA) | Gln (CAA) | Asn (AAC) | Asn (AAT) | Lys (AAA) | Tyr (TAT) |
| 2a | NGA/2018/dog/IZSSI_PA1464/19_idUV6-  MK895485 | Ala (GCA) | AGT (Ser) | Pro (CCA) | Gln (CAA) | Gln (CAA) | Gln (CAA) | Arg (AGA) | Val (GTA) | **Ala** (**G**CA) | Phe (TTT) | Arg (AGA) | Gln (CAA) | Asn (AAC) | Asn (AAT) | Lys (AAA) | Tyr (TAT) |
| 2a | NGA/2018/dog/IZSSI_PA1464/19_idUV1_TR_5A72-  MT840286 | Ala (GCA) | Ser (AGT) | Pro (CCA) | Gln (CAA) | Gln (CAA) | Gln (CAA) | Arg (AGA) | Val (GTA) | **Ala** (**G**CA) | Phe (TTT) | Arg (AGA) | Gln (CAA) | Asn (AAC) | Asn (AAT) | Lys (AAA) | Tyr (TAT) |
| 2a | NGA/2018/dog/616-  MN451689 | Ala (GCA) | Ser (AGT) | Pro (CCA) | Gln (CAA) | Gln (CAA) | Gln (CAA) | Arg (AGA) | Val (GTA) | **Ala** (**G**CA) | Phe (TTT) | Arg (AGA) | Gln (CAA) | Asn (AAC) | Asn (AAT) | Lys (AAA) | Tyr (TAT) |
| 2a | **NGA/2021/265.21-76-**  **ON0635455** | Ala (GCA) | AGT (Ser) | Pro (CCA) | Gln (CAA) | Gln (CAA) | Gln (CAA) | Arg (AGA) | Val (GTA) | **Ala** (**G**CA) | Phe (TTT) | Arg (AGA) | Gln (CAA) | Asn (AAC) | Asn (AAT) | Lys (AAA) | Tyr (TAT) |
| 2c | NGA/2018/dog/IZSSI_PA1464/19_idYV2 –  MK895486 | Ala (GCA) | AGT (Ser) | Pro (CCA) | Gln (CAA) | Gln (CAA) | **Arg** (C**G**A) | Arg (AGA) | Val (GTA) | Thr (ACA) | Phe (TTT) | Arg (AGA) | Gln (CAA) | Asn (AAC) | Asn (AAT) | Lys (AAA) | Tyr (TAT) |
| 2c | NGA/2018/dog/IZSSI_PA1464/19_idEV8-  MK895487 | Ala (GCA) | Ser (AGT) | Pro (CCA) | Gln (CAA) | Gln (CAA) | **Arg** (C**G**A) | Arg (AGA) | Val (GTA) | Thr (ACA) | Phe (TTT) | Arg (AGA) | Gln (CAA) | Asn (AAC) | Asn (AAT) | Lys (AAA) | Tyr (TAT) |
| 2c | NGA/2018/dog/IZSSI_PA1464/19_idJOE2-  MK895488 | **Gly** (G**G**A) | Ser (AGT) | Pro (CCA) | Gln (CAA) | Gln (CAA) | **Arg** (C**G**A) | Arg (AGA) | Val (GTA) | Thr (ACA) | Phe (TTT) | Arg (AGA) | Gln (CAA) | Asn (AAC) | Asn (AAT) | Lys (AAA) | Tyr (TAT) |
| 2c | NGA/2018/dog/IZSSI_PA1464/19_idNC-  MK895489 | **Gly** (G**G**A) | Ser (AGT) | Pro (CCA) | Gln (CAA) | Gln (CAA) | **Arg** (C**G**A) | Arg (AGA) | Val (GTA) | Thr (ACA) | Phe (TTT) | Arg (AGA) | Gln (CAA) | Asn (AAC) | Asn (AAT) | Lys (AAA) | Tyr (TAT) |
| 2c | NGA/2018/dog/IZSSI_PA1464/19_idPSV21-  MK895490 | Ala (GCA) | Ser (AGT) | Pro (CCA) | Gln (CAA) | Gln (CAA) | **Arg** (C**G**A) | Arg (AGA) | Val (GTA) | Thr (ACA) | Phe (TTT) | Arg (AGA) | Gln (CAA) | Asn (AAC) | Asn (AAT) | Lys (AAA) | Tyr (TAT) |
| 2c | NGA/2018/dog/IZSSI_PA1464/19_idJOE2_TR_4A72-  MT840287 | **Gly** (G**G**A) | Ser (AGT) | Pro (CCA) | Gln (CAA) | Gln (CAA) | **Arg** (C**G**A) | Arg (AGA) | Val (GTA) | Thr (ACA) | Phe (TTT) | Arg (AGA) | Gln (CAA) | Asn (AAC) | Asn (AAT) | Lys (AAA) | Tyr (TAT) |
| 2c | NGA/2018/dog/IZSSI_PA1464/19_idNC_TR_4A72-  MT840288 | **Gly** (G**G**A) | Ser (AGT) | Pro (CCA) | Gln (CAA) | Gln (CAA) | **Arg** (C**G**A) | Arg (AGA) | Val (GTA) | Thr (ACA) | Phe (TTT) | Arg (AGA) | Gln (CAA) | Asn (AAC) | Asn (AAT) | Lys (AAA) | Tyr (TAT) |
| 2c | NGA/2018/dog/IZSSI_PA1464/19_idPSV21_TR_4A72-MT840289 | Ala (GCA) | Ser (AGT) | Pro (CCA) | Gln (CAA) | Gln (CAA) | **Arg** (C**G**A) | Arg (AGA) | Val (GTA) | Thr (ACA) | Phe (TTT) | Arg (AGA) | Gln (CAA) | Asn (AAC) | Asn (AAT) | Lys (AAA) | Tyr (TAT) |
| 2c | NGA/2018/dog/IZSSI_PA1464/19_idEV8_TR_4A72-  MT840290 | **Gly** (G**G**A) | Ser (AGT) | Pro (CCA) | Gln (CAA) | Gln (CAA) | **Arg** (C**G**A) | Arg (AGA) | Val (GTA) | Thr (ACA) | Phe (TTT) | Arg (AGA) | Gln (CAA) | Asn (AAC) | Asn (AAT) | Lys (AAA) | Tyr (TAT) |
| 2c | NGA/2018/dog/IZSSI_PA1464/19_idEV5_TR_4A72-  MT840291 | Ala (GCA) | Ser (AGT) | Pro (CCA) | Gln (CAA) | Gln (CAA) | **Arg** (C**G**A) | Arg (AGA) | Val (GTA) | Thr (ACA) | Phe (TTT) | Arg (AGA) | Gln (CAA) | Asn (AAC) | Asn (AAT) | Lys (AAA) | Tyr (TAT) |
| 2c | NGA/2018/dog/IZSSI_PA1464/19_idN1_TR_4A72-  MT840292 | **Gly** (G**G**A) | Ser (AGT) | Pro (CCA) | Gln (CAA) | Gln (CAA) | **Arg** (C**G**A) | Arg (AGA) | Val (GTA) | Thr (ACA) | Phe (TTT) | Arg (AGA) | Gln (CAA) | Asn (AAC) | Asn (AAT) | Lys (AAA) | Tyr (TAT) |
| 2c | NGA/2018/dog/IZSSI_PA1464-  MT840293 | **Gly** (G**G**A**)** | Ser (AGT) | Pro (CCA) | Gln (CAA) | Gln (CAA) | **Arg** (C**G**A) | Arg (AGA) | Val (GTA) | Thr (ACA) | Phe (TTT) | Arg (AGA) | Gln (CAA) | Asn (AAC) | Asn (AAT) | Lys (AAA) | Tyr (TAT) |
| 2c | NGA/2018/dog/IZSSI_PA1464/19_idYV7_TR_4A72-  MT840294 | **Gly** (G**G**A) | Ser (AGT) | Pro (CCA) | Gln (CAA) | Gln (CAA) | **Arg** (C**G**A) | Arg (AGA) | Val (GTA) | Thr (ACA) | Phe (TTT) | Arg (AGA) | Gln (CAA) | Asn (AAC) | Asn (AAT) | Lys (AAA) | Tyr (TAT) |
| 2c | **NGA/2021/265.21-4-**  **ON063543** | **Gly** (G**G**A) | Ser (AGT) | Pro (CCA) | Gln (CAA) | Gln (CA**G**) | **Arg** (C**G**A) | Arg (AGA) | Val (GTA) | Thr (ACA) | Phe (TTT) | Arg (AGA) | Gln (CAA) | Asn (AAC) | Asn (AAT) | Lys (AAA) | Tyr (TAT) |
| 2c | **NGA/2021/265.21-5-**  **ON063544** | **Gly** (G**G**A) | Ser (AGT) | Pro (CCA) | Gln (CAA) | Gln (CA**G**) | **Arg** (C**G**A) | Arg (AGA) | Val (GTA) | Thr (ACA) | Phe (TTT) | Arg (AGA) | Gln (CAA) | Asn (AAC) | Asn (AAT) | Lys (AAA) | Tyr (TAT) |
| 2c | **NGA/2021/265.21-11-**  **ON0635445** | **Gly** (G**G**A) | Ser (AGT) | **Ala** (**G**CA) | Gln (CAA) | Gln (CA**G**) | **Arg** (C**G**A) | Arg (AGA) | Val (GTA) | Thr (ACA) | **Ile** (**A**TT) | Arg (AGA) | Gln (CAA) | Asn (AAC) | Asn (AAT) | Lys (AAA) | Tyr (TAT) |
| 2c | **NGA/2021/265.21-13-**  **ON0635446** | **Gly** (G**G**A) | **Gly** (**G**GT) | Pro (CCA) | Gln (CAA) | Gln (CA**G**) | **Arg** (C**G**A) | Arg (AGA) | **Ala** (G**C**A) | Thr (ACA) | Phe (TTT) | Arg (AGA) | Gln (CAA) | Asn (AAC) | Asn (AAT) | Lys (AAA) | Tyr (TAT) |
| 2c | **NGA/2021/265.21-18-**  **ON0635447** | **Gly** (G**G**A) | Ser (AGT) | Pro (CCA) | Gln (CAA) | Gln (CA**G**) | **Arg** (C**G**A) | Arg (AGA) | Val (GTA) | Thr (ACA) | Phe (TTT) | Arg (AGA) | **Pro** (C**C**A) | Asn (AAC) | **Lys** (AA**A**) | Lys (AAA) | Tyr (TAT) |
| 2c | **NGA/2021/265.21-28-**  **ON0635448** | **Gly** (G**G**A) | Ser (AGT) | Pro (CCA) | Gln (CAA) | Gln (CA**G**) | **Arg** (C**G**A) | Arg (AGA) | Val (GTA) | Thr (ACA) | Phe (TTT) | Arg (AGA) | Gln (CAA) | Asn (AAC) | Asn (AAT) | Lys (AAA) | Tyr (TAT) |
| 2c | **NGA/2021/265.21-42-**  **ON0635449** | **Gly** (G**G**A) | Ser (AGT) | Pro (CCA) | Gln (CAA) | Gln (CA**G**) | **Arg** (C**G**A) | Arg (AGA) | Val (GTA) | Thr (ACA) | Phe (TTT) | Arg (AGA) | Gln (CAA) | Asn (AAC) | Asn (AAT) | Lys (AAA) | Tyr (TAT) |
| 2c | **NGA/2021/265.21-58-**  **ON0635450** | **Gly** (G**G**A) | Ser (AGT) | Pro (CCA) | Gln (CAA) | Gln (CAA) | **Arg** (C**G**A) | Arg (AGA) | Val (GTA) | Thr (ACA) | Phe (TTT) | Arg (AGA) | Gln (CAA) | Asn (AAC) | Asn (AAT) | Lys (AAA) | Tyr (TAT) |
| 2c | **NGA/2021/265.21-59-**  **ON0635451** | **Gly** (G**G**A) | Ser (AGT) | Pro (CCA) | Gln (CAA) | Gln (CA**G**) | **Arg** (C**G**A) | Arg (AGA) | Val (GTA) | Thr (ACA) | Phe (TTT) | **Gly** (**G**GA) | Gln (CAA) | **Thr** (A**C**C) | Asn (AAT) | **Asn** (AA**T**) | **Phe** (T**T**T) |
| 2c | **NGA/2021/265.21-61-**  **ON0635452** | **Gly** (G**G**A) | Ser (AGT) | Pro (CCA) | Gln (CAA) | Gln (CA**G**) | **Arg** (C**G**A) | Arg (AGA) | Val (GTA) | Thr (ACA) | Phe (TTT) | Arg (AGA) | Gln (CAA) | Asn (AAC) | Asn (AAT) | Lys (AAA) | Tyr (TAT) |
| 2c | **NGA/2021/265.21-71-**  **ON0635453** | **Gly** (G**G**A) | Ser (AGT) | Pro (CCA) | Gln (CAA) | Gln (CA**G**) | **Arg** (C**G**A) | Arg (AGA) | Val (GTA) | Thr (ACA) | Phe (TTT) | Arg (AGA) | Gln (CAA) | Asn (AAC) | Asn (AAT) | Lys (AAA) | Tyr (TAT) |
| 2c | **NGA/2021/265.21-72-**  **ON0635454** | **Gly** (G**G**A) | Ser (AGT) | Pro (CCA) | Gln (CAA) | Gln (CA**G**) | **Arg** (C**G**A) | Arg (AGA) | Val (GTA) | Thr (ACA) | Phe (TTT) | Arg (AGA) | Gln (CAA) | Asn (AAC) | Asn (AAT) | Lys (AAA) | Tyr (TAT) |
| 2c | **NGA/2021/265.21-79-**  **ON0635456** | **Gly** (G**G**A) | Ser (AGT) | Pro (CCA) | **His** (CA**C**) | **His** (CA**C**) | **Arg** (C**G**A) | **Thr** (A**C**A) | Val (GTA) | Thr (ACA) | Phe (TTT) | Arg (AGA) | Gln (CAA) | Asn (AAC) | Asn (AAT) | Lys (AAA) | Tyr (TAT) |
| 2c | **NGA/2021/265.21-82-**  **ON0635457** | **Gly** (G**G**A) | Ser (AGT) | Pro (CCA) | Gln (CAA) | Gln (CA**G**) | **Arg** (C**G**A) | Arg (AGA) | Val (GTA) | Thr (ACA) | Phe (TTT) | Arg (AGA) | Gln (CAA) | Asn (AAC) | Asn (AAT) | Lys (AAA) | Tyr (TAT) |
| 2c | **NGA/2021/265.21-83-**  **ON0635458** | **Gly** (G**G**A) | Ser (AGT) | Pro (CCA) | Gln (CAA) | Gln (CA**G**) | **Arg** (C**G**A) | Arg (AGA) | Val (GTA) | Thr (ACA) | Phe (TTT) | Arg (AGA) | Gln (CAA) | Asn (AAC) | Asn (AAT) | Lys (AAA) | Tyr (TAT) |
| 2c | **NGA/2021/265.21-85-**  **ON0635459** | **Gly** (G**G**A) | Ser (AGT) | Pro (CCA) | Gln (CAA) | Gln (CA**G**) | **Arg** (C**G**A) | Arg (AGA) | Val (GTA) | Thr (ACA) | Phe (TTT) | Arg (AGA) | Gln (CAA) | Asn (AAC) | Asn (AAT) | Lys (AAA) | Tyr (TAT) |
| 2c | **NGA/2021/265.21-86-**  **ON0635460** | **Gly** (G**G**A) | Ser (AGT) | Pro (CCA) | Gln (CAA) | Gln (CA**G**) | **Arg** (C**G**A) | Arg (AGA) | Val (GTA) | Thr (ACA) | Phe (TTT) | Arg (AGA) | Gln (CAA) | Asn (AAC) | Asn (AAT) | Lys (AAA) | Tyr (TAT) |
| 2c | **NGA/2021/265.21-88-**  **ON0635461** | **Gly** (G**G**A) | Ser (AGT) | Pro (CCA) | Gln (CAA) | Gln (CA**G**) | **Arg** (C**G**A) | Arg (AGA) | Val (GTA) | Thr (ACA) | Phe (TTT) | Arg (AGA) | Gln (CAA) | Asn (AAC) | Asn (AAT) | Lys (AAA) | Tyr (TAT) |
| 2c | **NGA/2021/265.21-89-**  **ON0635462** | **Gly** (G**G**A) | Ser (AGT) | Pro (CCA) | Gln (CAA) | Gln (CA**G**) | **Arg** (C**G**A) | Arg (AGA) | Val (GTA) | Thr (ACA) | Phe (TTT) | Arg (AGA) | Gln (CAA) | Asn (AAC) | Asn (AAT) | Lys (AAA) | Tyr (TAT) |
| 2c | **NGA/2021/265.21-98-**  **ON0635463** | **Gly** (G**G**A) | Ser (AGT) | Pro (CCA) | Gln (CAA) | Gln (CA**G**) | **Arg** (C**G**A) | Arg (AGA) | Val (GTA) | Thr (ACA) | Phe (TTT) | Arg (AGA) | Gln (CAA) | Asn (AAC) | Asn (AAT) | Lys (AAA) | Tyr (TAT) |
| 2c | **NGA/2021/265.21-99-**  **ON0635464** | **Gly** (G**G**A) | Ser (AGT) | Pro (CCA) | Gln (CAA) | Gln (CA**G**) | **Arg** (C**G**A) | Arg (AGA) | Val (GTA) | Thr (ACA) | Phe (TTT) | Arg (AGA) | Gln (CAA) | Asn (AAC) | Asn (AAT) | Lys (AAA) | Tyr (TAT) |
